# Analysis of DNA Double-Strand Breaks and Cytotoxicity after 7 Tesla Magnetic Resonance Imaging of Isolated Human Lymphocytes

**DOI:** 10.1371/journal.pone.0132702

**Published:** 2015-07-15

**Authors:** Annika Reddig, Mahsa Fatahi, Björn Friebe, Karina Guttek, Roland Hartig, Frank Godenschweger, Dirk Roggenbuck, Jens Ricke, Dirk Reinhold, Oliver Speck

**Affiliations:** 1 Institute of Molecular and Clinical Immunology, Otto-von-Guericke-University Magdeburg, Magdeburg, Germany; 2 Department of Biomedical Magnetic Resonance, Otto-von-Guericke-University Magdeburg, Magdeburg, Germany; 3 Department of Radiology and Nuclear Medicine, Otto-von-Guericke-University Magdeburg, Magdeburg, Germany; 4 Medipan GmbH, Dahlewitz/Berlin, Germany; 5 Faculty of Natural Sciences, Brandenburg University of Technology Cottbus-Senftenberg, Senftenberg, Germany; 6 Leibniz Institute for Neurobiology, Magdeburg, Germany; 7 Center for Behavioral Brain Sciences, Magdeburg, Germany; 8 German Center for Neurodegenerative Disease, Magdeburg, Germany; National Research Council, ITALY

## Abstract

The global use of magnetic resonance imaging (MRI) is constantly growing and the field strengths increasing. Yet, only little data about harmful biological effects caused by MRI exposure are available and published research analyzing the impact of MRI on DNA integrity reported controversial results. This in vitro study aimed to investigate the genotoxic and cytotoxic potential of 7 T ultra-high-field MRI on isolated human peripheral blood mononuclear cells. Hence, unstimulated mononuclear blood cells were exposed to 7 T static magnetic field alone or in combination with maximum permissible imaging gradients and radiofrequency pulses as well as to ionizing radiation during computed tomography and γ-ray exposure. DNA double-strand breaks were quantified by flow cytometry and automated microscopy analysis of immunofluorescence stained γH2AX. Cytotoxicity was studied by CellTiter-Blue viability assay and [^3^H]-thymidine proliferation assay. Exposure of unstimulated mononuclear blood cells to 7 T static magnetic field alone or combined with varying gradient magnetic fields and pulsed radiofrequency fields did not induce DNA double-strand breaks, whereas irradiation with X- and γ-rays led to a dose-dependent induction of γH2AX foci. The viability assay revealed a time- and dose-dependent decrease in metabolic activity only among samples exposed to γ-radiation. Further, there was no evidence for altered proliferation response after cells were exposed to 7 T MRI or low doses of ionizing radiation (≤ 0.2 Gy). These findings confirm the acceptance of MRI as a safe non-invasive diagnostic imaging tool, but whether MRI can induce other types of DNA lesions or DNA double-strand breaks during altered conditions still needs to be investigated.

## Introduction

As technology advances, global emission of man-made electromagnetic fields (EMF) will further increase [1]. While there is proof that high energetic ionizing radiation, such as X-rays or γ-radiation, leads to DNA damage and carcinogenesis, uncertainties about health risks caused by non-ionizing radiation still remain [2]. The expanded exposure to non-ionizing radiation by power lines, various wireless communication devices or by the growing use of magnetic resonance imaging (MRI) has raised new safety concerns [3]. The lack of knowledge about EMF technologies on public health provoked the European Commission and the World Health Organization (WHO) to start different international research programs [4,5]. Furthermore, the WHO and International Commission on Non-Ionizing Radiation Protection (ICNIRP) in particular stated an urgent need to perform reliable studies analyzing short and long term adverse effects caused by MRI [6].

For MR image acquisition three different types of electromagnetic fields need to be combined: static magnetic field (SMF) at 0 Hz, gradient magnetic field (GMF) in the kHz frequency range and pulsed-radiofrequency field (RF) in the MHz range [1]. In order to improve signal-to-noise ratio and to shorten scanning time MRI scanners with higher SMF, stronger RF and faster GMF switching were constructed [7]. This development compulsory comes along with a higher energy deposition in the tissue. For RF fields energy deposition can be measured by the specific absorption rate (SAR), which increases with the square of the magnetic field strength if imaging methods are not adapted [8]. Until now especially ultrahigh-field-imaging (UHF), exceeding a SMF of 3 T, is impaired by high SAR, which constitutes one of the reasons, why UHF-MRI has not been implemented into routine diagnostic imaging yet [3,9].

While many studies have been performed analyzing the biological consequences of only a single type of electromagnetic field, little is published about their combined effect in MRI [1]. Reports discussing the genotoxic impact of high-field (HF)-MRI, with SMF between 1–3 T, are controversial and insufficient to draw any conclusion. Some publications reported an induction of DNA double-strand breaks (DSB) [10] or an increase in micronucleus formation [11] and comet tail moment [12], whereas others could not find significant changes in DNA integrity [13,14]. Even less data are available about genotoxic testing after UHF-MRI. For better risk estimation concerning patients and volunteers as well as occupationally exposed medical personnel, more knowledge about biological effects caused by HF- and UHF-MRI needs to be gained. In contrast to previous studies, we decided to use a 7 T SMF combined with the maximum permissible switched gradient and SAR. This experimental approach was used to show potential adverse effects of the electromagnetic fields used in MRI, as one would expect an increased impact on cell cytotoxicity and DSB formation due to these enhanced energy levels.

A sensitive biomarker for radiation biodosimetry is γH2AX [15,16]. Upon DSB formation, the core histone protein H2AX becomes rapidly phosphorylated at serin-139, termed γH2AX. Accumulation of γH2AX molecules at the break site allows visualization of an individual DSB as a single nuclear focus after immunofluorescence staining [17]. Among different immunological γH2AX tests, which are all based on the specific binding of an anti-γH2AX antibody, the microscopic immunofluorescence test is claimed to be the most sensitive method. It allows detection of individual cells and visualization of discrete γH2AX-foci [18]. However, manual counting of γH2AX-foci is time-consuming and subjective, therefore computational approaches have been developed [19,20]. One system for fully automated γH2AX foci evaluation is the AKLIDES platform, which has been used and evaluated by our laboratory and others in previous studies [19,21–23]. DSB are thought to be the most severe type of DNA lesion and various studies confirmed the suitability of γH2AX assay to determine a dose-dependent formation of DSB in blood lymphocytes after X-ray-based imaging, such as computed tomography (CT) or mammography [24–25].

The aim of the present in vitro study was to investigate the potential genotoxic and cytotoxic impact of 7 T UHF-MRI on human peripheral blood mononuclear cells (PBMCs) under defined and reproducible conditions. For genotoxicity testing γH2AX focus evaluation was carried out by automated fluorescence microscopy and flow cytometry measurements. For cytotoxicity assessments, proliferation analysis by [^3^H]-thymidine uptake in subsequently PHA-stimulated PBMCs and CellTiter-Blue viability assays were performed. In order to enhance the potential degree of damage and unlike in vivo protocols, MR echo planar imaging (EPI) sequences were adjusted to reach the maximum permissible gradient effect and a SAR of 100%. Our findings did not indicate a rise in DSB formation or induction of cytotoxicity neither after a 1 h exposure of unstimulated PBMCs to 7 T SMF alone nor in combination with extended EPI sequences.

## Material and Methods

### Ethics statement

The study was approved by the ethics committee of the Otto-von-Guericke University Magdeburg (RAD244 DSB-MRT) and healthy volunteers gave written informed consent.

### Cell culture

For lymphocyte isolation, 50 ml venous blood were obtained by venipuncture from 16 healthy donors each (8 male; 8 female; age 25–58 years, mean age 36 years). To prevent clotting, blood was directly added to 25 ml RPMI containing 5000 U/L heparin. The entire blood was prepared for PBMCs isolation, performing density gradient centrifugation. Therefore, two 50-ml conical polypropylene centrifuge tubes (Greiner Bio-One, Kremsmuenster, Austria) per donor were filled with 12.5 ml Biocoll separating solution (Biochrom, Berlin, Germany) and heparinized whole blood was carefully layered onto the Biocoll layer. Afterwards tubes were centrifuged at 490 x g for 30 min at 18°C with reduced breaks. Then the mononuclear cell layer was carefully transferred into a different centrifuge tube, cells were washed twice in RMPI 1640 medium (Biochrom) and resuspended to a final density of 1 x 10^6^ cells/ml in RPMI containing 10% fetal calf serum (FCS, Pan Biotech, Aidenbach, Germany), 100 U/ml penicillin and 100 μg/ml streptomycin (both Life Technologies GmbH, Darmstadt, Germany). For each donor six 50-ml conical centrifuge tubes were filled with 10 ml cell suspension each and kept at 37°C in a humidified atmosphere with 7% CO_2_ for approximately 30 min prior to exposure.

### Exposure conditions

The PBMC suspension of each donor was divided into 6 sample tubes according to the investigated exposure conditions. Cells were either i) left untreated, ii) exposed to 7 T SMF alone (7 T-B_0_) or iii) in combination with extended EPI sequences (7 T-EPI). Further, cells were irradiated by iv) X-ray-based CT scans and by γ-rays at a dose of v) 0.2 Gy or vi) 30 Gy. All MRI experiments were performed in a 1 h scan procedure inside a 7 T whole-body MR scanner (Siemens AG, Healthcare sector, Erlangen, Germany) equipped with a maximum gradient strength of 70 mT/m and a maximum gradient slew rate of 200 mT/m/ms. In contrast to protocols used for in vivo analysis, the EPI sequence (7900 ms TR, 22 ms TE, 80° flip angle, 0.8 mm × 0.8 mm × 1.5 mm voxel size, 100 slices) for these in vitro experiments was adjusted to the maximum permissible switched gradient and SAR.

MRI exposure was performed with an 8-channel head coil and in normal operating mode, in which no medical supervision is needed. In normal as well as first level controlled operating mode the maximum permissible head (local) average SAR is limited to 10 W/kg in 7 T MRI scanners. In the current study the SAR value was based on the local head average of a human head inside the scanner with an approximate weight of 5 kg. By adjusting the repetition time and flip angle the SAR limit was set close to the maximum permissible level for the head (10 W/kg). An average RF-power of 50 W was used. Further, the EPI pulse sequence applied a maximum gradient strength of 65.43 mT/m achieving a maximum slew rate of 186 mT/m/ms and a maximum readout gradient strength of 35.33 mT/m.

In each session four 50-ml conical polypropylene centrifuge tubes (Greiner Bio-One, Kremsmuenster, Austria), containing 10 ml cell solution, each from a different donor were arranged as a square in a test tube rack. This was placed inside the MRI scanner within a distance of the individual tubes of approximately 1 to 4 cm from the iso-center, where the RF field in the coil can be considered homogeneous and the gradients are in their linear regime. The untreated control samples were handled virtually in the same way as the MR-samples. Tubes were also carried to the MR building, but were placed in a different room at room temperature. Afterwards MR samples as well as control tubes were put on ice and carried back to the laboratory. No further analyses of incubator control samples were included in this study.

For comparison with ionizing radiation, cells were exposed to X-rays by conducting a spiral CT-scan (Aquilion Prime, Toshiba Medical Systems, Tustin, California, USA or Siemens Somatom Definition AS, Siemens Medical Systems, Erlangen, Germany, respectively) with a constant potential of 120 kV and a current of 200 mA with an aluminum filter of 3 mm (Toshiba) or 6.8 mm (Siemens), respectively and a rotation time of 0.5 seconds. These parameters led to a mean free air volumetric CT dose index (CTDI_air_) of 37.4 mGy with 5 mm collimated beam width. CTDI_air_ was supposed to be more appropriate than CTDI_vol_ as absorption of X-rays in this experimental setting was considered to be at a negligible low level. As positive controls, cells were radiated by γ-rays at a dose of 0.2 and 30 Gy (Biobeam 8000, Cs 137, Gamma-Service Medical GmbH, Leipzig, Germany). All exposures were performed at room temperature. Afterwards, tubes were put on ice for a maximum of 1.5 hours until treatment of remaining samples was completed.

### Detection of DNA double-strand breaks

For γH2AX analysis, cells were fixed at three different times. Initial DSB were measured in PBMCs stored on ice after exposure and fixed immediately (0 h) after treatment of all samples was finished. Additionally, cells were harvested after a 1 h incubation period, in order to allow phosphorylation at 37°C and 7% CO_2_. Further, cells were harvested 20 h after exposure to determine residual γH2AX foci.

For microscopy analysis, immunofluorescence staining was performed as described previously [19] with slight adjustments. In brief, PBMCs were washed in PBS, pipetted onto silanized glass slides and fixed for 15 min with 1% PFA. Slides with cells fixed immediately or 1 h after exposure were covered with PBS and kept overnight at 4°C. On the next day cells were harvested and fixed 20 h past exposure and staining of all slides was performed. After three washing steps in PBS cells were permeabilized in 0.2% Triton X-100 on ice and blocked with PBS containing 1% BSA. Subsequently, cells were stained with an anti-phosphohistone H2AX mouse monoclonal IgG primary antibody (Millipore, Schwalbach, Germany, clone JBW301, batch: 2310355) at a dilution of 1:2000 for 1 h at room temperature. Slides were washed and incubated with a 1:2000 diluted polyclonal goat anti-mouse IgG antibody conjugated to Alexa Fluor 488 (Lifetechnologies, Darmstadt, Germany, catalog number A11001, batch: 1298479) for 1 h at room temperature. After a final washing cycle in PBS, slides were covered with DAPI (4’,6-diamidino-2-phenylindole)-containing mounting medium (Medipan, Berlin/Dahlewitz, Germany).

On the same day of staining, slides were analyzed using fully automated γH2AX foci interpretation by the AKLIDES platform (Medipan), as described in detail elsewhere [19]. The system is based on a motorized inverse fluorescence microscope combined with different hard- and software modules. Fully automated image acquisition, analysis and evaluation of slides was performed in the blue channel to detect DAPI signals and in the green channel to analyze γH2AX foci using an objective with 60 x magnification (Olympus, Tokyo, Japan). The blue DAPI staining was applied for autofocusing and for the automated identification of cell nuclei. Furthermore, morphological parameters were implemented to exclude cell aggregates, granulocytes and the majority of monocytes. Hence, only single nuclei with a diameter between 4–10 μm, comprising the size of resting lymphocytes, were selected and involved in further analysis. Additionally, shape factors describing the circularity of an object were included. To reject elongated objects the threshold for the axis ratio, describing the ratio of maximum to minimum radius was empirically defined to be <1.3 for lymphocyte selection on the AKLIDES platform. The optimal range for the convexity of a nucleus was determined to be between 0.9–1.0, where a convexity of 1.0 describes a perfect circle and decreases depending on the concavities and indentations at the periphery of the object. To consider focus overlapping in z-stack images, γH2AX foci where analyzed in five different focal planes with 1 μm steps throughout each nucleus. The following parameters assessed from at least 200 cells per sample were used for further evaluation: mean γH2AX foci/cell, mean fluorescence intensity (MFI) of the nucleus in γH2AX channel as well as percentage of γH2AX focus-positive cells and classification of cells according to their individual focus number. Under the exposure conditions applied for image acquisition in γH2AX channel early apoptotic cells as well as cells treated with a very high dose of ionizing radiation (e.g. 30 Gy) show a pan-stained, overexposed nucleus. These cells were not further incorporated into γH2AX foci and intensity analysis but recorded separately as cells exhibiting a pan-nuclear staining.

For flow cytometry measurements, cells were stained in round bottomed falcon tubes analog to the protocol for immunohistochemistry preparation. For cytometry preparation, an additional fixation after PFA-treatment with 70% ethanol was included, according to the protocol of Redon et al. [18]. Therefore, cells harvested immediately or 1 h after exposure were stored in 70% ethanol overnight at 4°C. In contrast, cells fixed 20 h post exposure on the next day were treated with 70% ethanol for 20 min at room temperature. After permeabilization cells were either stained with anti-γH2AX antibody or IgG-isotype control. In order to enhance the intensity signal, the dilution of the secondary antibody was reduced from 1:2000 for microscopy to 1:500 for flow cytometry analysis. Stained samples were kept on ice in the dark until measurement. PBMCs were identified by forward and side scattered light signals and by an additional fluorescence signal originating from 0.5 μM DAPI (Sigma-Aldrich, St. Louis, MO, USA) staining. For flow cytometry measurements, a minimum of 20,000 gated events was analysed for each sample. Among the PBMC population the level of γH2AX was quantified by the median fluorescence intensity (MFI) in arbitrary units (AU) using a BD LSRFortessa cell analyzer (BD Biosciences, Mountain View, CA, USA) and FlowJo analyzing software (Treestar Inc., Ashland, OR, USA). For harmonization, MFI data were adjusted by subtraction of the corresponding IgG-isotype control of each donor, which was fixed together with the samples at time point 1 (0 h).

### Viability assay

To monitor the metabolic activity of unstimulated PBMCs, CellTiter-Blue assay (Promega, Madison, WI, USA) was performed according to the manufacturer’s instructions. In brief, 500 μl exposed cell suspension were diluted 1:1 with medium and 0.5 x 10^5^ cells/well were seeded as triplicates into flat bottomed 96-well plates. The cell concentration was adopted form the manufacturer’s instructions, stating a linear relationship between the fluorescence signal and the number of cells from 0 to 50,000 cells per well. CellTiter-Blue reagent resazurin was added to the wells either 24 h, 48 h or 84 h after exposure and plates were allowed to incubate in the dark for additional 2 h at 37°C and 7% CO_2_. The fluorescent signal was measured at an excitation of 560 nm and an emission of 590 nm by a Tecan Safire plate reader equipped with appropriate Magellan data analysis software (Tecan Austria GmbH, Salzburg, Austria). Cell viability was normalized to corresponding control samples.

### Proliferation assay

DNA synthesis of exposed cells was assessed by a standard [^3^H]-thymidine incorporation assay. Therefore, PBMCs were seeded at 1 x 10^5^ cells/well into a flat bottomed 96-well plate as quadruplicates and stimulated with 2 μg/ml phytohemagglutinin (PHA, life technologies/Gibco, UK). After 84 h, cells were pulsed with [^3^H]-thymidine at a dose of 0.2 μCi/well for additional 6 h. At the end of the incubation period cells were harvested and [^3^H]-thymidine incorporation was quantified using the microplate liquid scintillation counter Wallac MicroBeta TriLux from Perkin Elmer (Waltham, MA, USA).

### Statistical analysis

Quantitative results were statistically analyzed by GraphPad Prism software version 5.01 (Graph Pad Software, La Jolla, CA, USA). Significance levels were calculated by repeated measures ANOVA with 95% confidence interval (α = 0.05) followed by Dunnett’s post-hoc test, to compare the results after different exposure conditions to the data of the control group. Statistical analyses were only performed between data of the same assay at the same time point, according to the individual diagrams. No statistic testing was carried out among data of different experimental setups. Data are presented as the mean with standard deviation (mean ± SD) or mean with minimal and maximal value (range) within the text. In figures results are shown as mean with standard error of the mean (SEM) as error bars and P values are indicated by asterisks (***: P ≤ 0.001, **: P ≤ 0.01, *: P ≤ 0.05, not significant (ns): P > 0.05).

## Results

### Evaluation of γH2AX foci formation

**Fig 1:**
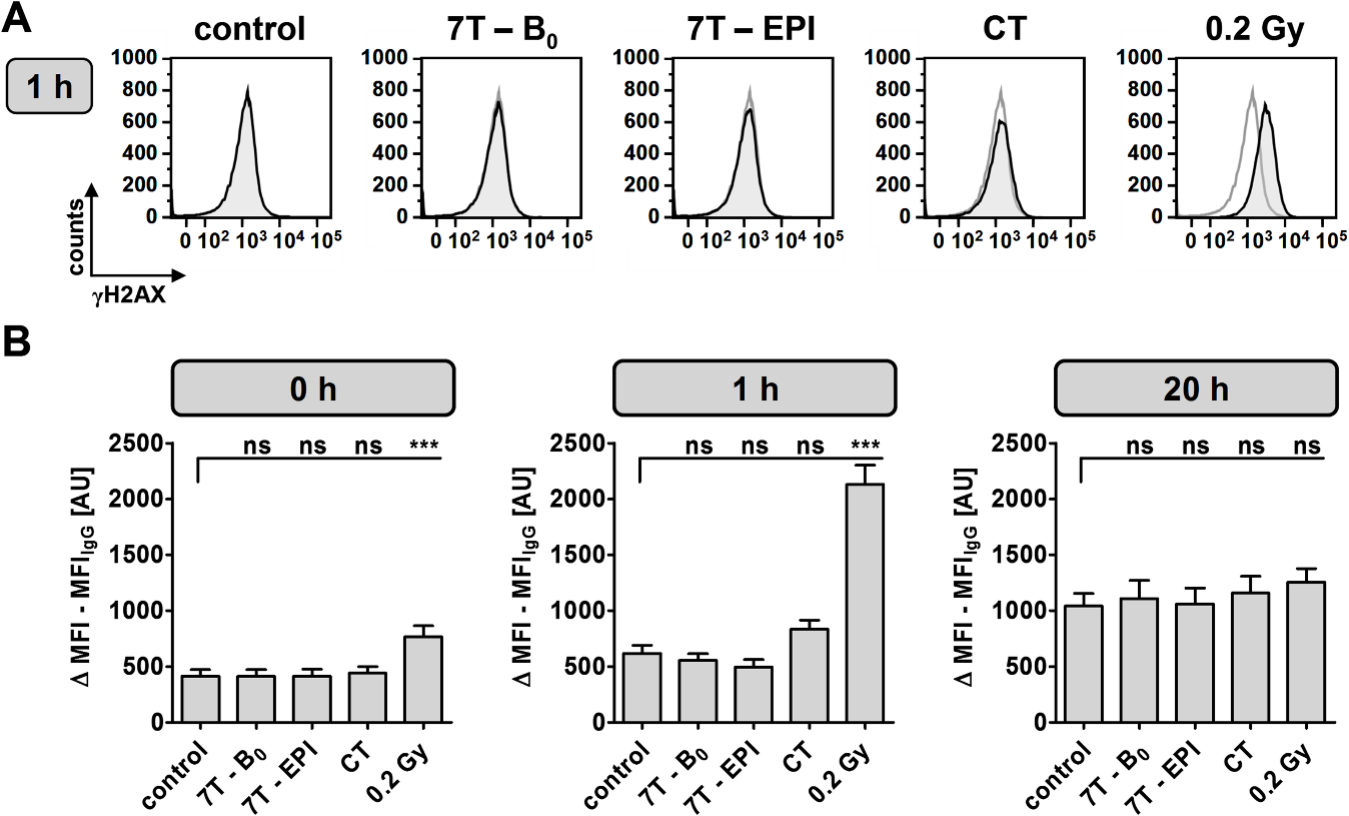
Flow cytometry analysis of γH2AX-stained DNA double-strand breaks. Mean γH2AX intensity was assessed in PBMCs immediately, 1 h and 20 h after indicated exposure conditions. (A) Representative overlay histogram of γH2AX-intensity 1 h after indicated exposure (black line) and of corresponding control (gray line). (B) Difference of mean fluorescence intensity (MFI) of γH2AX and IgG-isotype control staining from 16 independent experiments at three different time points after exposure as mean ± SEM (***: P ≤ 0.001; ns: P > 0.05).

MRI-induced DNA damage was studied by γH2AX immunofluorescence staining at three different time points. Results of flow cytometry analysis are shown in Fig 1 and S1 Table. Compared to control (MFI = 414 AU; range 38–807 AU) experiments revealed no changes in γH2AX intensity after exposure to 7 T SMF alone (MFI = 412 AU; range 12–927 AU) or after exposure to 7 T SMF combined with EPI (MFI = 414 AU; range 80–899 AU) at time point 0 h. Only a minor increase in γH2AX level could be detected directly after CT scan (MFI = 442 AU; range 65–960 AU), whereas a significant rise (MFI = 765 AU; range 184–1744 AU) was observed in 0.2 Gy γ-irradiated cells. Following an incubation period of 1 h at 37°C, cells further phosphorylated H2AX molecules, which was reflected by an enhanced intensity, especially among samples exposed to ionizing-radiation (CT: MFI = 834; range 155–1458 AU and 0.2 Gy: MFI = 2130 AU; range 1151–3378 AU). After 1 h the fluorescence intensity of the controls reached a MFI of 618 AU (range 67–1129 AU) and for cells exposed to 7 T SMF in the absence or presence of EPI a MFI of 556 AU (range 205–908 AU) and 498 AU (range: 50–917 AU) were observed, respectively. Due to DSB repair 20 h past exposure, the determined γH2AX levels in cells after 7 T and CT exposure did not differ significantly from control (MFI = 1042 AU; range 81–1807 AU) and even samples previously treated with 0.2 Gy γ-radiation only showed a slight increase in MFI (MFI = 1256 AU; range 103–1963 AU). The general intensity level of samples harvested after 20 h was higher compared to cells fixed on day one. This represents not only an increase in γH2AX foci but also reflects different fixation conditions. Internal experiments revealed that ethanol fixation overnight reduced autofluorescence, resulting in a higher background intensity level of cells fixed in 70% ethanol for only 20 min on the next day.

**Fig 2:**
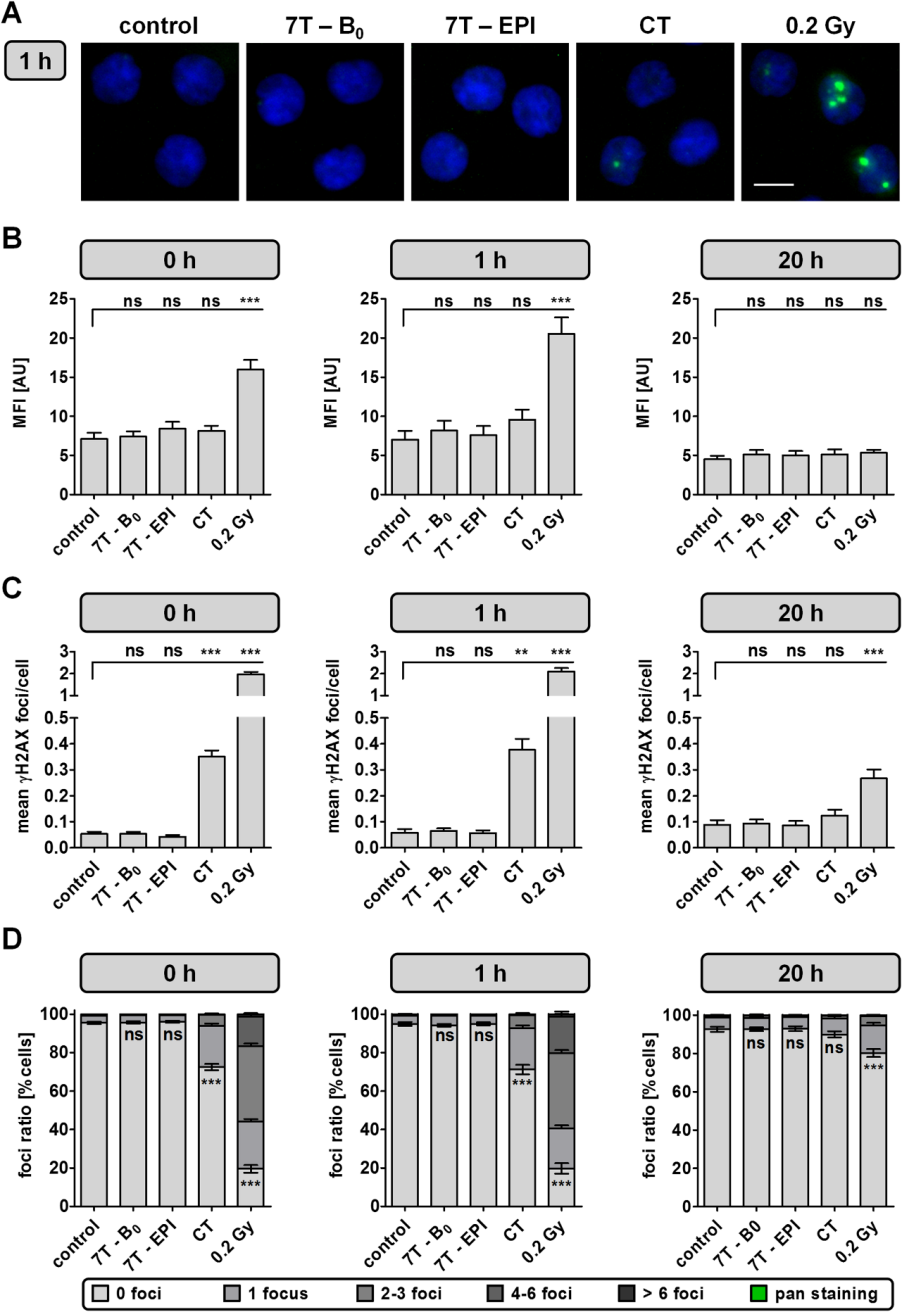
Analysis of γH2AX-stained DNA double-strand breaks by automated microscopy. γH2AX focus analysis was assessed in PBMCs immediately, 1 h and 20 h after indicated exposure conditions. (A) Representative images of DAPI (blue) and γH2AX-stained (green) PBMCs measured 1 h after indicated exposure. Bar: 5 μm. (B) Mean fluorescence intensity (MFI) of γH2AX-level, (C) amount of mean γH2AX foci/cell and (D) mean foci ratio from 16 independent experiments analyzed at three different time points after exposure as mean ± SEM (***: P ≤ 0.001; **: P ≤ 0.01; *: P ≤ 0.05; ns: P > 0.05). Cells with nuclei exhibiting the maximum γH2AX fluorescence signal throughout the whole nucleus were classified as pan-stained. These cells were recorded separately and not included into γH2AX focus and intensity analysis.

In parallel, γH2AX analysis was performed by automated microscopy (Fig 2, S2 Table, S3 Table, and S4 Table). Intensity data (Fig 2B) were similar to the results obtained by flow cytometry. For automated DSB quantification no differences in the mean number of γH2AX foci/cell between cells harvested immediately or 1 h after exposure were determined. Cells exposed to 7 T SMF alone (1 h—mean: 0.065 γH2AX foci/cell; range: 0.005–0.137) or 7 T SMF in combination with EPI sequences (1 h—mean: 0.057 γH2AX foci/cell; range: 0.004–0.169) revealed no changes in number of DSB compared to baseline level of unexposed cells (1 h—mean: 0.058 γH2AX foci/cell; range: 0.009–0.196) (Fig 2C). A significant (P < 0.01) rise in γH2AX foci formation was detected for all samples irradiated with X-rays during CT scan (1 h—mean: 0.377 γH2AX foci/cell; range: 0.183–0.730) and 0.2 Gy (mean: 2.101 γH2AX foci/cell; range: 1.063–3.123). Despite the reduced amount of DSB 20 h after irradiation, 0.2 Gy treated cells (mean: 0.267 γH2AX foci/cell; range: 0.101–0.542) still showed a statistically relevant increase in γH2AX foci, compared to the unexposed control (mean: 0.088 γH2AX foci/cell; range: 0.021–0.225).

Another parameter in γH2AX foci analysis is the ratio of DSB-positive and DSB-negative cells (Fig 2D). The results of γH2AX foci quantification are also reflected by the percentage of γH2AX positive cells. On average 5.0 ± 4.0% of untreated cells as well as 5.6 ± 2.9% and 4.9 ± 3.1% of cells exposed to 7 T SMF without or with combined EPI sequences were classified as γH2AX-positive, respectively. A significant decrease in γH2AX-foci negative cells was determined after exposure of PBMCs to ionizing radiation. Furthermore, the repair could be monitored by the foci ratio determined 20 h after exposure. Samples irradiated with 30 Gy were not included in γH2AX analysis, since 1 h after exposure 90.0 ± 5.8% of cells showed a pan-nuclear staining. Even though early apoptotic cells often show a pan-stained nucleus, this can only be interpreted as a hint for apoptosis. Due to fixed exposure times also cells containing a high amount of radiation-induced DSB can show a pan-nuclear, overexposed staining, which is reversible as DNA damage is repaired. In order to quantify the amount of apoptotic cells, a specific apoptosis marker needs to be applied.

### Analysis of cell viability by CellTiter-Blue assay

**Fig 3:**
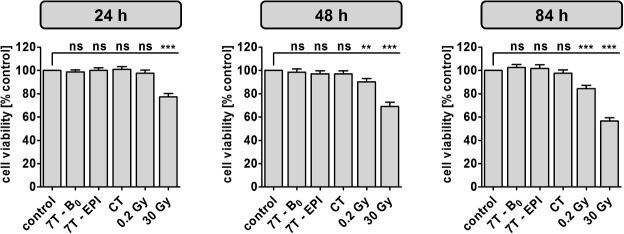
Cell viability analysis of unstimulated PBMCs by CellTiter-Blue assay. Metabolic activity was measured 24 h, 48 h and 84 h after indicated exposure conditions. Diagrams display mean ± SEM of 16 independent experiments (***: P ≤ 0.001; **: P ≤ 0.01; ns: P > 0.05).

For cytotoxicity analysis, cell viability of unstimulated PBMCs was determined 24 h, 48 h and 84 h after exposure (Fig 3 and S5 Table). After 24 h, a significant reduction in cell viability could only be detected in positive controls irradiated with 30 Gy, which showed a cell viability of 77.2 ± 11.9% compared to normalized control (100%). Viability of PBMCs exposed to γ-radiation was further reduced after 48 h to a level of 90.1% ± 11.8% at a dose of 0.2 Gy and 69.0% ± 15.1% at 30 Gy. After 84 h viability decreased to 84.4% ± 11.6% and 56.6% ± 11.8%, respectively. No significant changes in viability could be detected at any of the analyzed time points for PBMCs exposed to 7 T magnetic field or CT.

### Assessment of proliferation response by [^3^H]-thymidine incorporation

**Fig 4:**
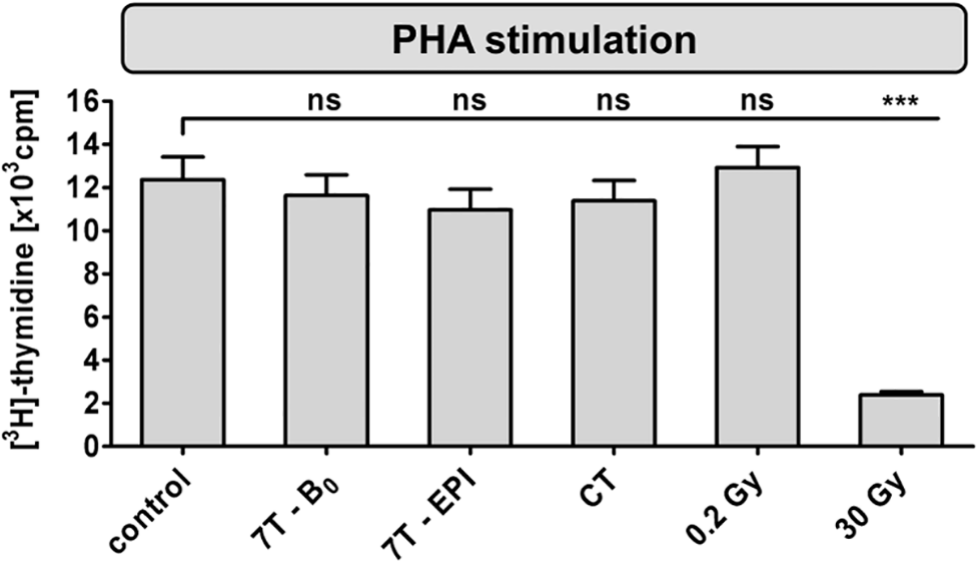
Proliferation assay of subsequently PHA-stimulated PBMCs. [^3^H]-thymidine incorporation was determined 84 h after indicated exposure conditions. Diagram displays mean ± SEM of 16 independent experiments (***: P ≤ 0.001; ns: P > 0.05).

Proliferation response was investigated 84 h after exposed PBMCs were subsequently stimulated with PHA (Fig 4 and S6 Table). Compared to unstimulated cells (497 ± 238 cpm), PHA-induced proliferation lead to a mean [^3^H]-thymidine incorporation of 12,362 ± 4,220 cpm in unexposed control samples. No significant changes in proliferation response were detected for cells exposed to 7 T SMF field alone (11,630 ± 3,849 cpm) or to 7 T SMF combined with GMF and RF in EPI sequence (10,967 ± 3,827 cpm). Further, a strong reduction to a proliferation rate of 19% was determined for cells irradiated with 30 Gy (2,382 ± 634 cpm). PBMCs exposed to lower doses of ionizing radiation, CT (11,386 ± 3,765 cpm) and 0.2 Gy (12,924 ± 3,895 cpm), did not differ significantly from control.

## Discussion

In contrast to ionizing radiation, which is known to induce DNA DSB even at very low doses, energy levels associated with electromagnetic fields used in MRI are not sufficient to induce direct breaking of chemical bonds [26]. But still, it cannot be excluded that indirect effects of EMF might harm DNA integrity. One possible mechanism is thought to be the generation of oxidative stress during MRI, which can lead to the formation of various DNA lesions [26,27]. DSB are described to be the most dangerous type of DNA damage, since they are difficult to repair and errors can lead to cell death, mutations and, tumorigenesis [28]. A fast and sensitive approach for DSB detection is the γH2AX-foci assay, with detection limits of radiation-induced DSB stated to be much lower compared to neutral comet assay or micronucleus test [20].

Recently, an in vivo study by Fiechter et al. [10] including 20 subjects reported an induction of DSB in lymphocytes after 1.5 T cardiac MRI. Imaging was conducted with standard pulse sequences and with a maximum gradient strength of 42 mT/m and a maximum gradient speed of 180 mT/m/ms. DSB were quantified by microscopy and flow cytometry analyses of immunofluorescence-stained γH2AX foci. However, before-and-after values show a high deviation. Furthermore, the source of DNA damage could not be distinguished between MRI-related effects, contrast agent effects and others, since sham exposed controls as well as patients receiving non-contrast enhanced MRI were not included. A significant increase in lymphocyte DNA damage, measured by alkaline comet assay, was also described by Yildiz et al. [29], analyzing 1.5 T hypophysial MRI of 28 subjects. Here, the maximum gradient field strength was 30 mT/m with a maximum slew rate of 125 T/m/s. Compared to baseline level non-contrast enhanced MRI revealed a small but not significant rise in DNA damage, whereas the amount of DNA lesions increased significantly under gadolinium-treated conditions. An additional in vitro study analyzing the impact of this contrast agent on human lymphocytes stated an enhanced cytotoxicity and genotoxicity by gadolinium treatment alone [30]. Furthermore, Simi et al. [11] reported a dose-dependent induction of micronuclei in human lymphocytes of 8 donors exposed in vitro to different MRI intensities as well as in vivo after non-contrast enhanced 1.5 T cardiac MRI scans of 8 subjects. The MR scanner was equipped with a maximum gradient strength of 50 mT/m and a maximum gradient speed of 150 mT/m/s. Analyzing one blood sample exposed to different exposure times of a routine 3 T MRI, Lee et al. [12] described a dose-dependent increase of micronuclei, chromosome aberration and comet tail moment. The estimated SAR of different pulse sequences that were applied ranged from 1.2 to 2.9 W/kg. In contrast to this study, no induction of DNA damage was observed in an equal in vitro trial, repeated by Szerencsi et al. [14], where SAR ranged from 1.2 to 3 W/kg. A study by Schwenzer et al. [13], utilizing γH2AX staining, reported no induction of DSB in two human leukemia cell lines (HL-60 and ATCC) 1 h and 24 h after exposure to 3 T MRI SMF alone as well as in 3 conditions applying 3 T MRI with different pulse sequences. Here, the maximum gradient strength was 40 mT/m and the maximum slew rate was 200 T/m/s. Depending on the sequence protocol estimated SAR rated from <0.1 W/kg to 4.1 W/kg.

In the current study, unstimulated human PBMCs were exposed to the SMF of a 7 T MRI alone or combined with imaging gradients and pulsed radiofrequency in EPI sequences. In order to investigate the ‘worst case scenario’ and enhance the potential degree of damage, the power deposited in samples was maximized by using the maximum permissible SAR level and switched gradient. In contrast, previous studies were more focused on commonly used sequences which do not necessarily include the maximum gradient and SAR level. For comparison, cells radiated by routine CT scan as well as γ-rays at doses of 0.2 Gy and 30 Gy were included. Genotoxicity assessment of γH2AX-stained cells did not reveal any differences in levels of DSB after 7 T exposures compared to untreated samples. Automated γH2AX-focus analysis determined a baseline number of 0.06 foci/cell in unexposed PBMCs, which compares well with levels reported by others [10,31]. Furthermore, radiation-induced samples showed a dose-dependent increase in DSB formation. Indeed, 1 h after CT exposure, a significant increase in scored γH2AX foci could be detected leading to an average of 0.38 foci/cell. Similar results have also been reported by Kuefner et al. [32]. Our experiments also display the repair of ionizing radiation induced DSB, which is reflected by a significant decline in γH2AX level 20 h post exposure. Further, γH2AX analysis also demonstrated different sensitivities according to the applied evaluation method. As stated previously, microscopy analysis allows the detection of even a single, small and low intensive γH2AX focus [18]. This can be the reason, why microscopy appeared to be more sensitive compared to flow cytometry within our study. Furthermore, no differences in metabolic activity between untreated, UHF-MRI and CT-exposed samples could be observed, whereas viability decreased over time and in a dose-dependent manner in γ-irradiated samples. Nevertheless, the applied cellTiter-Blue assay determines the reduction of resazurin to fluorescent resorufin in viable cells with active metabolism and interpretation of these results regarding cytotoxicity is limited. Combining this assay with additional parameters, analyzing membrane integrity and activity of apoptotic markers will provide more information about the number of dead cells and the mechanism of cell death. Analysis of proliferation response revealed no significant changes after UHF-MRI exposure. No significant changes in viability and proliferation response of unstimulated lymphocytes were also reported by different studies exposing cells to high magnetic fields. However, harmful effects due to strong magnetic fields were reported when lymphocytes were stimulated with PHA prior to exposure [33,34]. Further, we cannot conclude from this study, whether UHF leads to DNA damage in proliferating cells, generation of oxidative stress, or formation of other types of DNA lesions (e.g. base modifications, single-strand breaks), which in the long term might increase the risk to develop cancer.

With respect to MRI related genetic damage investigations, apart from comprehensive reviews by international organizations such as WHO, ICNIRP and SCENIHR (Scientific Committee on Emerging and Newly Identified Health Risks), there are seven peer reviewed reports available in the literature which are explained in details in the review paper by Vijayalaxmi et al. [35]. Some of those studies reported an induction of DNA damage whereas the others did not find any changes in DNA integrity. Taking into account that field strengths and exposure conditions differed among these studies it is difficult to compare their results and draw a consistent conclusion. In six studies human cells or cell lines were analyzed after exposure to either 1.5 or 3 T MRI, but no correlation between positive and negative finding according to the applied field strength, stated SAR or exposure time was found. Altogether, contradictory data do not need to result from different MR exposure conditions but can further be caused by heterogeneity in the experimental designs and methods applied in these studies. Whereas γH2AX analysis is a very sensitive measurement for DNA DSB quantification, alkaline comet assay also allows detection of DNA single-strand breaks [36]. Single-strand breaks are the most frequent type of DNA lesions in cells and occur spontaneously, or can be induced by exogenous physical and chemical agents [37]. Compared to direct γH2AX detection the MN assay examines a different end point and requires activation of cells for 72 h. Micronuclei can be formed during replication, which originate from induced DSB but also from inappropriate repair of different kinds of DNA lesions [38]. Reports comparing γH2AX, comet and/or MN assay have been published, showing consistencies of results but also individual limitations and specificities [39–42]. As a matter of fact, a gold standard method for genotoxicity determination after exposure to non-ionizing radiation has not been defined yet.

In conclusion, our in vitro results showed no increase in cytotoxicity and γH2AX foci formation after 7 T MRI but proved a significant induction of DSB after CT exposure. This confirms the acceptance of MRI as a safe imaging tool. Additional studies are in progress, investigating the genotoxic impact of MRI under in vivo conditions. However, according to the precautionary principle, an appropriate use of CT as well as MR imaging techniques should be ensured and the individual risk-benefit analysis between potential DNA damage and use of diagnostic imaging should be considered.

## Supporting Information

S1 Table Individual data depicted in Fig 1B: Mean fluorescence intensity (MFI) of γH2AX staining in PBMCs analysed by flow cytometry as arbitrary units [AU]. (DOC)

S2 Table Individual data depicted in Fig 2B: Mean fluorescence intensity (MFI) of γH2AX staining determined by automated microscopy as arbitrary units [AU]. (DOC)

S3 Table Individual data depicted in Fig 2C: Mean γH2AX foci/cell determined by automated microscopy. (DOC)

S4 Table Individual data depicted in Fig 2D: Mean percentage of γH2AX foci negative cells determined by automated microscopy. (DOC)

S5 Table Individual data depicted in Fig 3: Cell viability analysis of unstimulated PBMCs by CellTiter-Blue assay normalized to control (100%). (DOC)

S6 Table Individual data depicted in Fig 4: Proliferation of PBMCs in cpms determined by [3H]-thymidine incorporation after 84 h. (DOC)
