# Supplementary material for: Analysis of DNA Double-Strand Breaks and Cytotoxicity after 7 Tesla Magnetic Resonance Imaging of Isolated Human Lymphocytes
Source: PLoS One. 2015 Jul 15;10(7):e0132702. doi: 10.1371/journal.pone.0132702 (PMC4503586; doi:10.1371/journal.pone.0132702)
Supplement: S1 Table — (DOC) [file pone.0132702.s001.doc]

**S1 Table. Individual data depicted in Figure 1b: Mean fluorescence intensity (MFI) of γH2AX staining in PBMCs analysed by flow cytometry as arbitrary units [AU].**

|  | **MFI** | **Δ MFI-MFI_IgG_ [AU]** | | | | | | | | | | | | | | | |
| --- | --- | --- | --- | --- | --- | --- | --- | --- | --- | --- | --- | --- | --- | --- | --- | --- | --- |
|  | **0 h** | **0 h** | | | | | **1 h** | | | | | **20 h** | | | | | |
| **Donor No.** | **IgG** | **control** | **7T-B_0_** | **7T-EPI** | **CT** | **0.2 Gy** | **control** | **7T-B_0_** | **7T-EPI** | **CT** | **0.2 Gy** | **control** | | **7T-B_0_** | **7T-EPI** | **CT** | **0.2 Gy** |
| **01** | 531 | 807 | 803 | 889 | 960 | 1744 | 804 | 853 | 588 | 993 | 2727 | 1246 | 1652 | | 1540 | 1487 | 1416 |
| **02** | 443 | 494 | 322 | 318 | 439 | 690 | 443 | 323 | 374 | 759 | 2084 | 707 | 748 | | 802 | 828 | 764 |
| **03** | 454 | 665 | 643 | 625 | 723 | 750 | 589 | 488 | 602 | 767 | 1820 | 1255 | 1351 | | 1313 | 1378 | 1407 |
| **04** | 537 | 469 | 461 | 570 | 543 | 847 | 497 | 492 | 465 | 637 | 2193 | 1106 | 1160 | | 1133 | 1045 | 1027 |
| **05** | 2275 | 643 | 469 | 500 | 612 | 1199 | 871 | 908 | 908 | 1458 | 3179 | 1807 | 1722 | | 1805 | 1670 | 1606 |
| **06** | 2147 | 278 | 307 | 311 | 615 | 765 | 753 | 680 | 596 | 1070 | 2488 | 1206 | 1136 | | 1154 | 1147 | 1214 |
| **07** | 2156 | 254 | 228 | 376 | 367 | 627 | 866 | 205 | 206 | 717 | 2918 | 923 | 921 | | 846 | 568 | 994 |
| **08** | 1860 | 115 | 12 | 117 | 231 | 852 | 460 | 470 | 328 | 684 | 3378 | 1322 | 833 | | 1037 | 1021 | 1316 |
| **09** | 1345 | 529 | 445 | 376 | 452 | 1330 | 1129 | 842 | 600 | 1079 | 2133 | 1317 | 1978 | | 1850 | 1612 | 1194 |
| **10** | 1316 | 326 | 380 | 146 | 263 | 930 | 480 | 333 | 89 | 155 | 1151 | 1771 | 1098 | | 1102 | 1417 | 1632 |
| **11** | 1783 | 164 | 149 | 899 | 151 | 319 | 714 | 644 | 599 | 428 | 1440 | 1053 | 2431 | | 1820 | 1945 | 1963 |
| **12** | 1666 | 564 | 927 | 479 | 563 | 670 | 1017 | 805 | 851 | 1252 | 2543 | 1056 | 1375 | | 1389 | 2129 | 890 |
| **13** | 571 | 317 | 296 | 292 | 434 | 487 | 432 | 632 | 429 | 663 | 1544 | 692 | 627 | | 583 | 719 | 1939 |
| **14** | 775 | 744 | 544 | 525 | 495 | 595 | 610 | 693 | 917 | 891 | 1454 | 423 | 296 | | 391 | 1460 | 1752 |
| **15** | 1022 | 38 | 430 | 114 | 65 | 184 | 67 | 249 | 365 | 611 | 1496 | 81 | 4 | | 43 | 65 | 103 |
| **16** | 1064 | 222 | 179 | 80 | 158 | 256 | 158 | 283 | 50 | 1181 | 1526 | 713 | 442 | | 170 | 43 | 886 |
| **mean** |  | **414** | **412** | **414** | **442** | **765** | **618** | **556** | **498** | **834** | **2130** | **1042** | **1111** | | **1061** | **1158** | **1256** |
| **std** |  | **233** | **238** | **252** | **234** | **404** | **288** | **233** | **263** | **329** | **685** | **451** | **638** | | **565** | **604** | **483** |
| **min** |  | **38** | **12** | **80** | **65** | **184** | **67** | **205** | **50** | **155** | **1151** | **81** | **4** | | **43** | **43** | **103** |
| **max** |  | **807** | **927** | **899** | **960** | **1744** | **1129** | **908** | **917** | **1458** | **3378** | **1807** | **2431** | | **1850** | **2129** | **1963** |
