# Supplementary material for: Analysis of DNA Double-Strand Breaks and Cytotoxicity after 7 Tesla Magnetic Resonance Imaging of Isolated Human Lymphocytes
Source: PLoS One. 2015 Jul 15;10(7):e0132702. doi: 10.1371/journal.pone.0132702 (PMC4503586; doi:10.1371/journal.pone.0132702)
Supplement: S2 Table — (DOC) [file pone.0132702.s002.doc]

**S2 Table. Individual data depicted in Figure 2b: Mean fluorescence intensity (MFI) of γH2AX staining determined by automated microscopy as arbitrary units [AU].**

|  | **0 h** | | | | | **1 h** | | | | | **20 h** | | | | |
| --- | --- | --- | --- | --- | --- | --- | --- | --- | --- | --- | --- | --- | --- | --- | --- |
| **Donor**  **No.** | **control** | **7T-B_0_** | **7T-EPI** | **CT** | **0.2 Gy** | **control** | **7T-B_0_** | **7T-EPI** | **CT** | **0.2 Gy** | **control** | **7T-B_0_** | **7T-EPI** | **CT** | **0.2 Gy** |
| **01** | 9.29 | 5.83 | 6.58 | 8.35 | 18.36 | 6.45 | 3.92 | 3.25 | 6.58 | 17.19 | 4.57 | 3.87 | 2.42 | 3.82 | 3.97 |
| **02** | 2.00 | 5.22 | 4.63 | 6.32 | 12.58 | 2.15 | 4.39 | 4.85 | 4.67 | 12.88 | 1.36 | 1.04 | 1.78 | 1.21 | 4.28 |
| **03** | 4.11 | 4.41 | 4.16 | 6.58 | 9.53 | 2.05 | 2.80 | 2.21 | 2.96 | 12.33 | 2.10 | 1.98 | 2.26 | 2.61 | 2.38 |
| **04** | 3.15 | 3.64 | 4.02 | 4.08 | 12.50 | 2.53 | 3.22 | 4.02 | 6.40 | 16.82 | 1.31 | 1.63 | 1.00 | 1.99 | 3.00 |
| **05** | 13.80 | 14.32 | 13.46 | 14.35 | 27.08 | 12.57 | 12.58 | 12.91 | 15.09 | 31.54 | 5.64 | 5.22 | 6.00 | 7.40 | 6.47 |
| **06** | 6.51 | 5.24 | 7.55 | 10.04 | 16.62 | 2.37 | 4.53 | 7.36 | 7.81 | 22.02 | 4.99 | 6.09 | 6.22 | 4.25 | 5.37 |
| **07** | 8.83 | 8.77 | 9.88 | 6.37 | 17.14 | 6.22 | 6.09 | 4.88 | 11.55 | 26.33 | 4.14 | 5.50 | 5.45 | 4.71 | 6.74 |
| **08** | 7.77 | 8.78 | 10.57 | 6.89 | 12.94 | 3.45 | 2.79 | 1.59 | 4.86 | 15.85 | 5.13 | 3.78 | 3.84 | 4.03 | 5.25 |
| **09** | 6.58 | 8.22 | 9.80 | 10.55 | 18.56 | 11.14 | 13.76 | 11.28 | 9.00 | 12.66 | 4.57 | 5.36 | 7.60 | 9.69 | 6.83 |
| **10** | 5.80 | 7.28 | 4.27 | 5.90 | 9.69 | 7.46 | 6.18 | 5.73 | 8.06 | 13.85 | 5.76 | 5.04 | 3.75 | 3.58 | 6.40 |
| **11** | 8.14 | 7.05 | 11.13 | 9.96 | 11.36 | 4.86 | 8.06 | 7.45 | 5.86 | 19.00 | 6.69 | 8.79 | 9.06 | 11.26 | 7.68 |
| **12** | 2.89 | 5.89 | 3.95 | 7.03 | 10.66 | 4.58 | 4.55 | 4.54 | 6.33 | 14.49 | 3.81 | 7.27 | 5.61 | 5.41 | 5.76 |
| **13** | 10.40 | 8.84 | 11.71 | 11.39 | 18.22 | 15.07 | 15.87 | 19.77 | 22.97 | 43.16 | 5.33 | 4.95 | 7.06 | 4.88 | 5.26 |
| **14** | 7.49 | 7.95 | 8.29 | 6.26 | 19.61 | 14.54 | 14.52 | 10.40 | 13.30 | 18.96 | 6.48 | 8.06 | 5.53 | 5.96 | 5.46 |
| **15** | 9.98 | 7.80 | 10.47 | 9.92 | 23.04 | 8.93 | 16.76 | 10.89 | 16.10 | 28.95 | 6.35 | 5.93 | 5.35 | 5.05 | 6.15 |
| **16** | 7.10 | 9.41 | 14.48 | 6.08 | 17.40 | 8.29 | 10.68 | 10.29 | 11.58 | 22.72 | 3.90 | 7.44 | 7.16 | 5.85 | 4.54 |
| **mean** | **7.12** | **7.42** | **8.43** | **8.13** | **15.96** | **7.04** | **8.17** | **7.59** | **9.57** | **20.55** | **4.51** | **5.12** | **5.01** | **5.11** | **5.35** |
| **std** | **3.10** | **2.53** | **3.52** | **2.66** | **4.97** | **4.39** | **5.03** | **4.76** | **5.25** | **8.43** | **1.70** | **2.25** | **2.30** | **2.62** | **1.43** |
| **min** | **2.00** | **3.64** | **3.95** | **4.08** | **9.53** | **2.05** | **2.79** | **1.59** | **2.96** | **12.33** | **1.31** | **1.04** | **1.00** | **1.21** | **2.38** |
| **max** | **13.80** | **14.32** | **14.48** | **14.35** | **27.08** | **15.07** | **16.76** | **19.77** | **22.97** | **43.16** | **6.69** | **8.79** | **9.06** | **11.26** | **7.68** |
