# Supplementary material for: Analysis of DNA Double-Strand Breaks and Cytotoxicity after 7 Tesla Magnetic Resonance Imaging of Isolated Human Lymphocytes
Source: PLoS One. 2015 Jul 15;10(7):e0132702. doi: 10.1371/journal.pone.0132702 (PMC4503586; doi:10.1371/journal.pone.0132702)
Supplement: S3 Table — (DOC) [file pone.0132702.s003.doc]

**S3 Table. Individual data depicted in Figure 2c: Mean γH2AX foci/cell determined by automated microscopy.**

|  | **0 h** | | | | | **1 h** | | | | | **20 h** | | | | |
| --- | --- | --- | --- | --- | --- | --- | --- | --- | --- | --- | --- | --- | --- | --- | --- |
| **Donor**  **No.** | **control** | **7T-B_0_** | **7T-EPI** | **CT** | **0.2 Gy** | **control** | **7T-B_0_** | **7T-EPI** | **CT** | **0.2 Gy** | **control** | **7T-B_0_** | **7T-EPI** | **CT** | **0.2 Gy** |
| **01** | 0.048 | 0.044 | 0.032 | 0.340 | 1.716 | 0.196 | 0.110 | 0.112 | 0.537 | 2.676 | 0.225 | 0.227 | 0.222 | 0.291 | 0.324 |
| **02** | 0.035 | 0.030 | 0.025 | 0.389 | 2.219 | 0.027 | 0.053 | 0.035 | 0.197 | 1.468 | 0.040 | 0.051 | 0.034 | 0.023 | 0.145 |
| **03** | 0.091 | 0.057 | 0.036 | 0.380 | 1.761 | 0.024 | 0.037 | 0.064 | 0.336 | 2.005 | 0.066 | 0.069 | 0.055 | 0.070 | 0.183 |
| **04** | 0.019 | 0.040 | 0.009 | 0.289 | 1.922 | 0.013 | 0.034 | 0.028 | 0.234 | 2.080 | 0.021 | 0.039 | 0.005 | 0.061 | 0.181 |
| **05** | 0.065 | 0.069 | 0.079 | 0.495 | 2.585 | 0.159 | 0.137 | 0.169 | 0.730 | 3.123 | 0.200 | 0.190 | 0.122 | 0.273 | 0.411 |
| **06** | 0.075 | 0.059 | 0.060 | 0.529 | 2.396 | 0.009 | 0.049 | 0.032 | 0.653 | 2.986 | 0.189 | 0.165 | 0.203 | 0.289 | 0.542 |
| **07** | 0.028 | 0.005 | 0.015 | 0.274 | 2.019 | 0.101 | 0.121 | 0.063 | 0.540 | 2.726 | 0.108 | 0.171 | 0.211 | 0.152 | 0.467 |
| **08** | 0.015 | 0.040 | 0.015 | 0.181 | 1.406 | 0.032 | 0.043 | 0.004 | 0.330 | 2.297 | 0.072 | 0.045 | 0.030 | 0.087 | 0.355 |
| **09** | 0.055 | 0.075 | 0.025 | 0.392 | 1.936 | 0.060 | 0.047 | 0.044 | 0.278 | 1.259 | 0.025 | 0.062 | 0.081 | 0.059 | 0.201 |
| **10** | 0.009 | 0.063 | 0.018 | 0.206 | 1.505 | 0.013 | 0.044 | 0.023 | 0.250 | 1.714 | 0.042 | 0.038 | 0.004 | 0.103 | 0.254 |
| **11** | 0.057 | 0.020 | 0.059 | 0.328 | 1.354 | 0.036 | 0.029 | 0.040 | 0.183 | 1.063 | 0.035 | 0.067 | 0.052 | 0.077 | 0.105 |
| **12** | 0.018 | 0.039 | 0.013 | 0.329 | 1.505 | 0.035 | 0.047 | 0.017 | 0.207 | 1.541 | 0.035 | 0.048 | 0.034 | 0.027 | 0.101 |
| **13** | 0.108 | 0.147 | 0.081 | 0.451 | 2.512 | 0.096 | 0.132 | 0.110 | 0.483 | 2.512 | 0.170 | 0.133 | 0.147 | 0.171 | 0.395 |
| **14** | 0.117 | 0.047 | 0.060 | 0.309 | 2.132 | 0.022 | 0.005 | 0.044 | 0.316 | 1.700 | 0.053 | 0.057 | 0.052 | 0.175 | 0.284 |
| **15** | 0.086 | 0.085 | 0.090 | 0.435 | 2.778 | 0.055 | 0.078 | 0.048 | 0.411 | 2.534 | 0.058 | 0.064 | 0.060 | 0.068 | 0.209 |
| **16** | 0.029 | 0.034 | 0.063 | 0.286 | 1.872 | 0.053 | 0.075 | 0.075 | 0.352 | 1.929 | 0.077 | 0.079 | 0.053 | 0.063 | 0.121 |
| **mean** | **0.053** | **0.053** | **0.042** | **0.351** | **1.976** | **0.058** | **0.065** | **0.057** | **0.377** | **2.101** | **0.088** | **0.094** | **0.085** | **0.124** | **0.267** |
| **std** | **0.034** | **0.032** | **0.027** | **0.097** | **0.436** | **0.054** | **0.040** | **0.042** | **0.167** | **0.622** | **0.068** | **0.062** | **0.073** | **0.091** | **0.136** |
| **min** | **0.009** | **0.005** | **0.009** | **0.181** | **1.354** | **0.009** | **0.005** | **0.004** | **0.183** | **1.063** | **0.021** | **0.038** | **0.004** | **0.023** | **0.101** |
| **max** | **0.117** | **0.147** | **0.090** | **0.529** | **2.778** | **0.196** | **0.137** | **0.169** | **0.730** | **3.123** | **0.225** | **0.227** | **0.222** | **0.291** | **0.542** |
