# Supplementary material for: Analysis of DNA Double-Strand Breaks and Cytotoxicity after 7 Tesla Magnetic Resonance Imaging of Isolated Human Lymphocytes
Source: PLoS One. 2015 Jul 15;10(7):e0132702. doi: 10.1371/journal.pone.0132702 (PMC4503586; doi:10.1371/journal.pone.0132702)
Supplement: S4 Table — (DOC) [file pone.0132702.s004.doc]

**S4 Table. Individual data depicted in Figure 2d: Mean percentage of γH2AX foci negative cells determined by automated microscopy.**

|  | **0 h** | | | | | **1 h** | | | | | **20 h** | | | | |
| --- | --- | --- | --- | --- | --- | --- | --- | --- | --- | --- | --- | --- | --- | --- | --- |
| **Donor**  **No.** | **control** | **7T-B_0_** | **7T-EPI** | **CT** | **0.2 Gy** | **control** | **7T-B_0_** | **7T-EPI** | **CT** | **0.2 Gy** | **control** | **7T-B_0_** | **7T-EPI** | **CT** | **0.2 Gy** |
| **01** | 94.7 | 97.6 | 96.8 | 72.8 | 24.6 | 86.4 | 91.5 | 91.0 | 63.4 | 13.6 | 82.4 | 84.5 | 84.1 | 77.3 | 76.2 |
| **02** | 97.5 | 97.0 | 97.5 | 67.6 | 14.9 | 97.3 | 94.7 | 96.5 | 81.6 | 29.1 | 96.5 | 94.9 | 96.6 | 97.7 | 89.3 |
| **03** | 93.5 | 94.8 | 96.8 | 71.2 | 21.5 | 98.1 | 96.8 | 95.4 | 71.9 | 18.2 | 94.2 | 94.0 | 94.5 | 93.5 | 85.3 |
| **04** | 98.1 | 96.0 | 99.1 | 76.3 | 22.0 | 98.7 | 96.6 | 97.2 | 79.4 | 24.0 | 98.3 | 96.6 | 99.5 | 94.4 | 84.4 |
| **05** | 94.5 | 94.6 | 91.7 | 63.1 | 12.5 | 86.6 | 90.3 | 87.9 | 53.2 | 5.4 | 86.5 | 88.3 | 88.9 | 79.9 | 70.6 |
| **06** | 93.4 | 94.1 | 94.8 | 59.6 | 11.7 | 98.6 | 95.1 | 96.8 | 54.9 | 9.2 | 84.8 | 87.7 | 85.4 | 79.4 | 64.7 |
| **07** | 97.7 | 99.5 | 98.1 | 77.6 | 16.1 | 90.9 | 89.7 | 93.7 | 59.6 | 8.7 | 90.9 | 87.6 | 85.0 | 86.2 | 69.6 |
| **08** | 99.0 | 97.5 | 98.5 | 83.7 | 30.9 | 96.8 | 94.8 | 99.6 | 71.0 | 14.0 | 94.3 | 96.5 | 97.0 | 92.6 | 73.4 |
| **09** | 96.5 | 95.0 | 98.5 | 70.3 | 17.1 | 94.8 | 95.3 | 95.2 | 76.5 | 36.8 | 97.9 | 95.2 | 92.5 | 94.5 | 86.3 |
| **10** | 98.6 | 96.9 | 98.2 | 82.1 | 28.1 | 98.7 | 96.5 | 97.7 | 80.0 | 23.9 | 95.8 | 96.7 | 99.6 | 91.2 | 79.5 |
| **11** | 95.8 | 98.0 | 95.1 | 75.1 | 34.5 | 96.4 | 96.6 | 97.0 | 83.3 | 43.2 | 96.1 | 93.3 | 94.8 | 92.8 | 90.5 |
| **12** | 98.2 | 96.9 | 97.8 | 75.4 | 27.1 | 96.5 | 97.2 | 98.3 | 82.3 | 29.7 | 96.6 | 95.2 | 96.7 | 97.3 | 91.8 |
| **13** | 92.7 | 89.0 | 93.2 | 66.2 | 12.4 | 92.8 | 89.9 | 90.7 | 63.4 | 8.7 | 85.6 | 90.2 | 88.5 | 88.2 | 71.6 |
| **14** | 88.8 | 95.8 | 94.5 | 76.1 | 10.3 | 97.8 | 99.0 | 95.6 | 77.1 | 18.5 | 95.6 | 94.3 | 94.8 | 86.5 | 78.5 |
| **15** | 92.7 | 93.4 | 93.8 | 66.3 | 8.7 | 94.5 | 93.2 | 95.7 | 68.8 | 9.7 | 94.7 | 94.6 | 94.9 | 93.3 | 82.6 |
| **16** | 96.7 | 96.6 | 93.7 | 77.2 | 19.7 | 94.7 | 92.5 | 92.9 | 74.2 | 22.9 | 92.0 | 92.1 | 94.7 | 93.7 | 88.8 |
| **mean** | **95.5** | **95.8** | **96.1** | **72.5** | **19.5** | **95.0** | **94.4** | **95.1** | **71.3** | **19.7** | **92.6** | **92.6** | **93.0** | **89.9** | **80.2** |
| **std** | **2.8** | **2.4** | **2.3** | **6.7** | **7.8** | **4.0** | **2.9** | **3.1** | **9.8** | **11.0** | **5.1** | **3.8** | **5.1** | **6.4** | **8.4** |
| **min** | **88.8** | **89.0** | **91.7** | **59.6** | **8.7** | **86.4** | **89.7** | **87.9** | **53.2** | **5.4** | **82.4** | **84.5** | **84.1** | **77.3** | **64.7** |
| **max** | **99.0** | **99.5** | **99.1** | **83.7** | **34.5** | **98.7** | **99.0** | **99.6** | **83.3** | **43.2** | **98.3** | **96.7** | **99.6** | **97.7** | **91.8** |
