# Supplementary material for: Analysis of DNA Double-Strand Breaks and Cytotoxicity after 7 Tesla Magnetic Resonance Imaging of Isolated Human Lymphocytes
Source: PLoS One. 2015 Jul 15;10(7):e0132702. doi: 10.1371/journal.pone.0132702 (PMC4503586; doi:10.1371/journal.pone.0132702)
Supplement: S5 Table — (DOC) [file pone.0132702.s005.doc]

**S5 Table. Individual data depicted in Figure 3: Cell viability analysis of unstimulated PBMCs by CellTiter-Blue assay normalized to control (100%).**

|  | **24 h** | | | | | | **48 h** | | | | | | **84 h** | | | | | |
| --- | --- | --- | --- | --- | --- | --- | --- | --- | --- | --- | --- | --- | --- | --- | --- | --- | --- | --- |
| **Donor**  **No.** | **control** | **7T-B_0_** | **7T-EPI** | **CT** | **0.2 Gy** | **30 Gy** | **control** | **7T-B_0_** | **7T-EPI** | **CT** | **0.2 Gy** | **30 Gy** | **control** | **7T-B_0_** | **7T-EPI** | **CT** | **0.2 Gy** | **30 Gy** |
| **01** | 100 | 100 | 100 | 88 | 86 | 72 | 100 | 108 | 94 | 81 | 82 | 60 | 100 | 100 | 103 | 72 | 68 | 43 |
| **02** | 100 | 95 | 101 | 106 | 95 | 93 | 100 | 111 | 104 | 111 | 92 | 82 | 100 | 107 | 100 | 103 | 79 | 58 |
| **03** | 100 | 105 | 105 | 100 | 100 | 77 | 100 | 112 | 104 | 99 | 98 | 64 | 100 | 114 | 107 | 93 | 89 | 50 |
| **04** | 100 | 108 | 109 | 104 | 97 | 67 | 100 | 110 | 103 | 93 | 83 | 50 | 100 | 121 | 114 | 87 | 69 | 41 |
| **05** | 100 | 113 | 105 | 109 | 108 | 96 | 100 | 110 | 117 | 122 | 122 | 114 | 100 | 103 | 102 | 96 | 91 | 65 |
| **06** | 100 | 111 | 107 | 113 | 113 | 77 | 100 | 112 | 112 | 105 | 101 | 71 | 100 | 117 | 116 | 110 | 102 | 75 |
| **07** | 100 | 104 | 120 | 119 | 127 | 99 | 100 | 102 | 102 | 99 | 101 | 77 | 100 | 105 | 99 | 96 | 95 | 70 |
| **08** | 100 | 96 | 96 | 90 | 98 | 75 | 100 | 93 | 92 | 92 | 95 | 73 | 100 | 113 | 109 | 104 | 99 | 75 |
| **09** | 100 | 93 | 99 | 108 | 91 | 73 | 100 | 85 | 93 | 101 | 80 | 66 | 100 | 89 | 97 | 107 | 86 | 52 |
| **10** | 100 | 93 | 92 | 91 | 89 | 68 | 100 | 85 | 86 | 86 | 79 | 62 | 100 | 98 | 96 | 102 | 87 | 64 |
| **11** | 100 | 86 | 78 | 89 | 87 | 72 | 100 | 84 | 68 | 78 | 77 | 62 | 100 | 79 | 61 | 73 | 67 | 43 |
| **12** | 100 | 100 | 104 | 106 | 103 | 92 | 100 | 96 | 96 | 97 | 95 | 80 | 100 | 106 | 108 | 107 | 97 | 66 |
| **13** | 100 | 95 | 91 | 92 | 87 | 60 | 100 | 93 | 89 | 89 | 79 | 52 | 100 | 98 | 98 | 96 | 72 | 42 |
| **14** | 100 | 93 | 97 | 99 | 96 | 81 | 100 | 94 | 93 | 97 | 89 | 69 | 100 | 97 | 100 | 103 | 88 | 61 |
| **15** | 100 | 92 | 97 | 101 | 93 | 63 | 100 | 92 | 99 | 102 | 86 | 59 | 100 | 100 | 111 | 110 | 88 | 51 |
| **16** | 100 | 94 | 98 | 100 | 91 | 70 | 100 | 90 | 100 | 99 | 83 | 63 | 100 | 91 | 106 | 103 | 73 | 49 |
| **mean** | **100.0** | **98.6** | **99.9** | **100.9** | **97.6** | **77.2** | **100.0** | **98.6** | **97.0** | **96.9** | **90.1** | **69.0** | **100.0** | **102.4** | **101.7** | **97.6** | **84.4** | **56.6** |
| **std** | **0.0** | **7.6** | **9.2** | **9.2** | **10.9** | **11.9** | **0.0** | **10.5** | **11.2** | **10.9** | **11.8** | **15.1** | **0.0** | **10.9** | **12.4** | **11.6** | **11.6** | **11.8** |
| **min** | **100.0** | **86.0** | **78.0** | **88.0** | **86.0** | **60.0** | **100.0** | **84.0** | **68.0** | **78.0** | **77.0** | **50.0** | **100.0** | **79.0** | **61.0** | **72.0** | **67.0** | **41.0** |
| **max** | **100.0** | **113.0** | **120.0** | **119.0** | **127.0** | **99.0** | **100.0** | **112.0** | **117.0** | **122.0** | **122.0** | **114.0** | **100.0** | **121.0** | **116.0** | **110.0** | **102.0** | **75.0** |
