# Supplementary material for: Analysis of DNA Double-Strand Breaks and Cytotoxicity after 7 Tesla Magnetic Resonance Imaging of Isolated Human Lymphocytes
Source: PLoS One. 2015 Jul 15;10(7):e0132702. doi: 10.1371/journal.pone.0132702 (PMC4503586; doi:10.1371/journal.pone.0132702)
Supplement: S6 Table — (DOC) [file pone.0132702.s006.doc]

**S6 Table. Individual data depicted in Figure 4: Proliferation of PBMCs in cpms determined by [^3^H]-thymidine incorporation after 84 h.**

|  | **unstimulated** | | | | | | **PHA-stimulation** | | | | | |
| --- | --- | --- | --- | --- | --- | --- | --- | --- | --- | --- | --- | --- |
| **Donor**  **No.** | **control** | **7T-B_0_** | **7T-EPI** | **CT** | **0.2 Gy** | **30 Gy** | **control** | **7T-B_0_** | **7T-EPI** | **CT** | **0.2 Gy** | **30 Gy** |
| **01** | 1118 | 1166 | 944 | 1055 | 1241 | 869 | 12151 | 13229 | 9836 | 9842 | 12332 | 2842 |
| **02** | 332 | 464 | 451 | 446 | 610 | 409 | 6222 | 6305 | 5770 | 7041 | 8050 | 3830 |
| **03** | 639 | 642 | 771 | 514 | 581 | 302 | 11012 | 10087 | 11033 | 10727 | 10405 | 2192 |
| **04** | 365 | 296 | 185 | 311 | 281 | 155 | 10080 | 8849 | 9872 | 10211 | 10548 | 2493 |
| **05** | 580 | 585 | 473 | 359 | 391 | 248 | 6934 | 7168 | 6556 | 6550 | 7989 | 2364 |
| **06** | 703 | 577 | 401 | 357 | 465 | 215 | 19405 | 18933 | 18433 | 19736 | 21121 | 3071 |
| **07** | 419 | 320 | 312 | 348 | 305 | 125 | 12173 | 10786 | 10997 | 11548 | 12624 | 2347 |
| **08** | 266 | 273 | 279 | 254 | 254 | 83 | 12867 | 12805 | 10149 | 10679 | 11853 | 1925 |
| **09** | 357 | 348 | 225 | 228 | 146 | 53 | 13052 | 12766 | 11452 | 12636 | 12993 | 1465 |
| **10** | 550 | 458 | 409 | 342 | 335 | 80 | 18013 | 16664 | 16199 | 14757 | 17978 | 3043 |
| **11** | 236 | 224 | 177 | 209 | 186 | 111 | 6939 | 8040 | 5780 | 5821 | 7313 | 1519 |
| **12** | 451 | 376 | 222 | 368 | 266 | 68 | 10739 | 9359 | 8994 | 9531 | 10649 | 2172 |
| **13** | 218 | 191 | 191 | 185 | 213 | 102 | 8017 | 6617 | 7093 | 8608 | 15004 | 2279 |
| **14** | 367 | 273 | 392 | 377 | 321 | 36 | 17709 | 16595 | 16188 | 16661 | 17010 | 2841 |
| **15** | 538 | 360 | 315 | 318 | 296 | 60 | 16969 | 14543 | 13911 | 14243 | 16601 | 1573 |
| **16** | 817 | 574 | 384 | 529 | 544 | 71 | 15503 | 13326 | 13202 | 13589 | 14313 | 2163 |
| **mean** | **497** | **445** | **383** | **388** | **402** | **187** | **12362** | **11630** | **10967** | **11386** | **12924** | **2382** |
| **std** | **238** | **237** | **211** | **203** | **263** | **209** | **4220** | **3849** | **3827** | **3765** | **3895** | **634** |
| **min** | **218** | **191** | **177** | **185** | **146** | **36** | **6222** | **6305** | **5770** | **5821** | **7313** | **1465** |
| **max** | **1118** | **1166** | **944** | **1055** | **1241** | **869** | **19405** | **18933** | **18433** | **19736** | **21121** | **3830** |
